# The value of cardiac magnetic resonance delayed enhancement combined with tissue tracking in discriminating cardiac amyloidosis from hypertrophic cardiomyopathy

**DOI:** 10.3389/fcvm.2025.1712928

**Published:** 2025-12-19

**Authors:** Xiao-Gang Xue, Xiao-Yong Xu, Xue-Yao Lin, Gao-Yan Wang, Hai-Bo Dong

**Affiliations:** 1 Department of Radiology, Ningbo Medical Center Lihuili Hospital, Ningbo, Zhejiang Province, China; 2 Department of Cardiology, Ningbo Medical Center Lihuili Hospital, Ningbo, Zhejiang Province, China

**Keywords:** amyloidosis, hypertrophic cardiomyopathy, magnetic resonance imaging, ventricular function, medicine

## Abstract

**Purpose:**

Cardiac amyloidosis (CA) and hypertrophic cardiomyopathy (HCM) may both present with left ventricular hypertrophy, making differential diagnosis challenging. This study aimed to evaluate the value of cardiac magnetic resonance (CMR) delayed enhancement combined with tissue tracking (CMR-TT) in discriminating CA from HCM.

**Methods:**

Data from 30 patients with CA, 29 patients with HCM, and 20 normal controls (NC) were retrospectively analyzed. All subjects underwent CMR examinations. Tissue tracking techniques were adopted for CMR cine sequences to directly quantify global radial strain (GRS), global circumferential strain (GCS), and global longitudinal strain (GLS).

**Results:**

The most common delayed enhancement pattern in CA was linear subendocardial enhancement (76.7%). Half of the CA patients had delayed enhancement involving atria and right ventricle, while 33.3% exhibited the characteristic “chaotic sign.” The GRS and GCS values were significantly different between the CA group and the HCM group and between the CA group and the NC group (*P* < 0.05). GLS differed significantly among the CA, HCM, and NC groups (*P* < 0.05). ROC analysis revealed that GCS (AUC = 0.748, *P* = 0.001) and GLS (AUC = 0.732, *P* = 0.002) provided good diagnostic efficiency in differentiating CA from HCM.

**Conclusion:**

CMR delayed enhancement patterns combined with myocardial strain parameters, particularly GLS and GCS, can aid in the differential diagnosis of CA and HCM. Optimal cutoff values of GCS (−14.6%) and GLS (−9.22%) provide a noninvasive imaging approach with significant clinical implications for guiding treatment and improving prognosis.

## Introduction

Cardiac amyloidosis (CA) is a myocardial disease caused by the progressive and infiltrative deposition of amyloid protein in the heart, leading to ventricular wall thickening, diastolic dysfunction, arrhythmias, and ultimately heart failure (1, 2). Hypertrophic cardiomyopathy (HCM), by contrast, is a genetic disease characterized by myocardial hypertrophy, often associated with myofibrillar disarray, interstitial fibrosis, and impaired relaxation. Despite their distinct pathophysiological mechanisms, CA and HCM share the common feature of left ventricular hypertrophy and may present with overlapping clinical manifestations such as dyspnea, chest pain, and arrhythmias. These similarities contribute to a substantial diagnostic challenge, with reports suggesting that up to 35% of CA cases are initially misdiagnosed as HCM (3). Such misdiagnoses can delay appropriate treatment and are associated with poorer outcomes, including significantly higher mortality rates in untreated or late-diagnosed CA patients (4). Therefore, improving the accuracy and timeliness of differential diagnosis is of great clinical significance.

Cardiac magnetic resonance imaging (CMR) provides unique advantages in the noninvasive assessment of cardiomyopathies, with its high soft tissue resolution, multiparametric imaging capability, and excellent reproducibility (5, 6). Late gadolinium enhancement (LGE) sequences can reveal patterns of myocardial infiltration, scarring, and fibrosis, which are valuable for differentiating between cardiomyopathies (7). Meanwhile, cardiac magnetic resonance tissue tracking (CMR-TT) has emerged as a novel technique that enables quantification of myocardial deformation in three dimensions—radial, circumferential, and longitudinal—based on cine sequences, thereby providing sensitive functional markers of myocardial damage (8–11). Previous studies have primarily focused on either LGE or strain parameters alone, but the diagnostic accuracy remains suboptimal. To date, few investigations have combined these two complementary modalities to enhance the differentiation between CA and HCM, representing an important research gap.

Accordingly, the present study aims to evaluate the value of combining CMR delayed enhancement with tissue tracking in distinguishing CA from HCM. Specifically, we sought to (i) characterize the delayed enhancement patterns in CA compared with HCM, (ii) analyze differences in global myocardial strain parameters among CA, HCM, and normal controls, and (iii) assess the diagnostic efficiency of strain indices in differentiating CA from HCM. By addressing this gap, we expect to provide a noninvasive, practical, and accurate imaging strategy to support earlier diagnosis, reduce misdiagnosis-related adverse outcomes, and guide timely therapeutic decision-making in clinical practice.

## Methods

### Clinical data

This retrospective study included 30 patients diagnosed with CA and 29 patients diagnosed with hypertrophic cardiomyopathy (HCM) at Ningbo Medical Center Lihuili Hospital from January 2019 to December 2023. Additionally, 20 age- and sex-matched healthy controls (NC) were recruited from health check-up volunteers during the same period. The inclusion criteria were as follows: (1) for the CA group, patients met the diagnostic criteria (12), including either confirmation of amyloid deposition in endomyocardial biopsy, or echocardiographic features highly suggestive of CA—such as diffuse thickening of both ventricular walls, impaired systolic function, and pericardial or pleural effusion—combined with biopsy-proven amyloidosis in extracardiac organs; among them, 25 patients had light chain amyloidosis (AL type) and 5 had transthyretin amyloidosis (ATTR type); (2) for the HCM group, the diagnostic criteria (13) included a maximal interventricular septum thickness ≥15 mm, or maximal left ventricular wall thickness ≥13 mm with a definite family history of HCM, measured at the thickest segment on standard short-axis cine images, with secondary causes of hypertrophy such as hypertension or valvular heart disease excluded; (3) for the NC group, eligible volunteers were required to have no history of congenital heart disease, hypertension, coronary artery disease, atherosclerosis, dyslipidemia, or other structural heart diseases. The exclusion criteria were: (1) secondary myocardial hypertrophy; (2) coexistence of other structural heart diseases; and (3) incomplete clinical or imaging data.

### CMR examination protocol

All subjects underwent CMR examination using a GE Discovery MR750 3.0T magnetic resonance scanner (GE Healthcare, Milwaukee, WI, USA) with a 16-channel phased-array cardiac coil. Imaging was performed according to the standardized cardiac planes and nomenclature recommended by the American Heart Association. Patients were scanned in the supine position with retrospective electrocardiogram gating and respiratory gating at end-expiration.

Cine sequences were acquired using fast imaging employing steady-state acquisition (FIESTA) sequences, covering the left ventricular short-axis, two-chamber, four-chamber, and long-axis planes from the base to the apex. The key imaging parameters were: slice thickness 8 mm, inter-slice gap 2 mm, TR 3.5 ms, TE 1.5 ms, flip angle (FA) 45°, field of view 360 mm × 280 mm, matrix 216 × 256, and temporal resolution 40 ms.

Contrast-enhanced CMR included first-pass perfusion and late gadolinium enhancement (LGE). Gadopentetate dimeglumine (GD-DTPA, Bayer Schering Pharma, Berlin, Germany) was administered intravenously at 0.2 mmol/kg body weight at a rate of 3–5 mL/s, followed by 20 mL normal saline. LGE imaging was performed 15 min post-injection using an inversion-recovery sequence, with inversion time individually adjusted (350–380 ms) to null normal myocardium. Twelve short-axis slices covering the entire left ventricle were obtained with parameters identical to cine imaging.

A specific “dark blood pool” phenomenon was also assessed as a potential diagnostic marker. This feature is characterized by loss of the normally bright blood pool signal on short-axis views, appearing as a dark cavity with reduced contrast between the ventricular wall and cavity, sometimes accompanied by the “chaotic sign.”

### Image postprocessing

**Figure 1:**
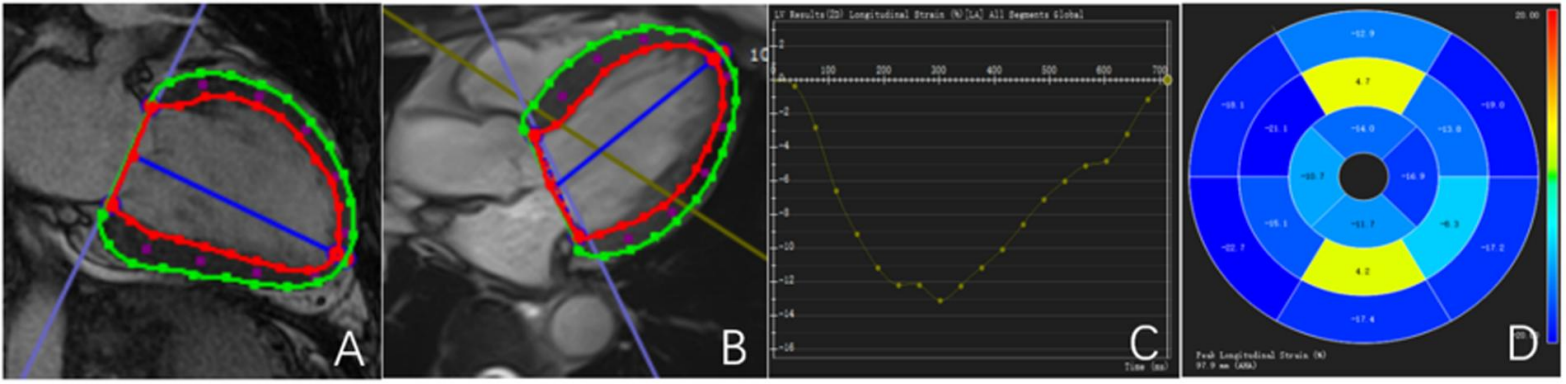
Outline and description of myocardial strain. **(A,B)** Left ventricular endocardial and epicardial strain curves are delineated; **(C)** left ventricular GLS curve; **(D)** left ventricular GLS bull's-eye maps. The red line represents the contour of the endocardium.The green line represents the outline of the epicardium.The blue line represents the central axis of the left ventricular cavity.

All CMR data were transferred to a dedicated workstation and analyzed using CVI 42 postprocessing software (version 5.6, Circle Cardiovascular Imaging Inc., Calgary, Alberta, Canada). Endocardial and epicardial contours of the left ventricle were manually traced at end-diastole and end-systole to calculate left ventricular ejection fraction (LVEF) and left ventricular myocardial mass (LVMM). Global myocardial deformation was evaluated using CMR tissue tracking (CMR-TT), with automatic voxel-based tracking throughout the cardiac cycle. Global radial strain (GRS), global circumferential strain (GCS), and global longitudinal strain (GLS) were measured, and corresponding strain curves and bull's-eye plots were generated (Figure 1).

To ensure measurement consistency, all analyses were performed by one experienced reader using a standardized protocol. Inter- and intra-observer reproducibility (ICC) was not assessed because each parameter was measured once; this limitation is acknowledged in the Discussion.

### Sample size estimation

Given the rarity of CA and HCM, sample size determination was based on previous literature and traditional estimation methods rather than formal power calculation. The present study aimed to include as many eligible patients as possible within the study period to ensure sufficient statistical validity.

### Statistical analysis

Statistical analyses were performed using SPSS software (version 25.0, IBM Corp., Armonk, NY, USA). Continuous variables were tested for normality. Normally distributed variables are expressed as mean ± standard deviation, and between-group comparisons were performed using independent sample *t*-tests or one-way analysis of variance (ANOVA). *Post-hoc* pairwise comparisons following ANOVA were corrected for multiple testing using the Bonferroni method. Non-normally distributed data are presented as medians (interquartile ranges) and compared using the Mann–Whitney *U* test or Kruskal–Wallis test as appropriate. Categorical variables are presented as frequencies and analyzed using the chi-square test.

Receiver operating characteristic (ROC) curve analysis was used to assess the diagnostic performance of strain indices in differentiating CA from HCM, and the area under the curve (AUC) was reported with 95% confidence intervals (CIs). The optimal cutoff value for each parameter was determined using the Youden index, defined as J = sensitivity + specificity − 1, where the maximum J indicates the point that best balances sensitivity and specificity. A two-sided *P* < 0.05 was considered statistically significant.

## Results

### Comparison of clinical baseline data

The CA group included 30 patients, with 17 males and an average age of 58.83 ± 8.89 years. The HCM group included 29 patients, with 16 males and an average age of 58.17 ± 8.53 years. The NC group consisted of 20 participants, with 10 males and an average age of 59.75 ± 7.73 years. There were no statistically significant differences in age or sex among the three groups.

The LVEF in the CA group was lower than that in the HCM and NC groups (*P* < 0.001), but there was no significant difference between the HCM group and the NC group. Compared with the NC group, both the CA and HCM groups presented significant increases in the LVMM (*P* < 0.001). However, there was no significant difference in myocardial mass between the CA and HCM groups.

In the CA group, 21 patients (70%) presented with left ventricular myocardial hypertrophy, among whom 16 presented with symmetric myocardial hypertrophy (53.3%). In the HCM group, all 29 patients (100%) presented with left ventricular myocardial hypertrophy, of whom 7 displayed symmetric myocardial hypertrophy (24.1%). While both the CA and HCM groups included cases of myocardial hypertrophy, the incidence was significantly greater in the HCM group (*P* < 0.001). Additionally, compared with the HCM group, the CA group presented symmetric and uniform left ventricular myocardial hypertrophy (*P* < 0.05).

The CA group included 17 patients (56.7%) with pericardial effusion and 15 patients (50%) with pleural effusion, whereas the HCM group included 4 patients (13.8%) with pericardial effusion and 3 patients (10.3%) with pleural effusion. The incidence of pericardial and pleural effusion in the CA group was significantly greater than that in the HCM group (*P* < 0.001) (Table 1).

**Table 1:** Comparison of clinical baseline data.

Variable	CA (*n* = 30)	HCM (*n* = 29)	NC (*n* = 20)	*P*
Age (years)	58.83 ± 8.89	58.17 ± 8.53	59.75 ± 7.73	0.789
Male (%)	17 (56.7%)	16 (55.2%)	10 (50%)	0.894
LVEF (%)	48.30 ± 11.54^a^^,^^c^	61.17 ± 7.77^b^	60.05 ± 3.22	<0.001
LVMM (g/m^2^)	79.89 ± 26.43^c^	81.62 ± 28.86^c^	44.40 ± 9.99	<0.001
Myocardial hypertrophy	21 (70%)	29 (100%)	0	<0.001
Symmetric myocardial hypertrophy	16 (53.3%)	7 (24.1%)	0	<0.05
Pericardial effusion	17 (56.7%)	4 (13.8%)	0	<0.001
Pleural effusion	15 (50%)	3 (10.3%)	0	<0.001

CA, cardiac amyloidosis; HCM, hypertrophic cardiomyopathy; NC, healthy control; LVEF, left ventricular ejection fraction; LVMM, left ventricular myocardial mass.

Data are presented as mean ± standard deviation or number (percentage).

a *P* < 0.05 vs. HCM group.

b *P* < 0.05 vs. NC group.

c *P* < 0.05 vs. CA group.

### Comparison of delayed enhancement patterns and extents

Both the CA and HCM groups exhibited various patterns of delayed enhancement in the left ventricular myocardium, including no enhancement, subendocardial enhancement, mid-myocardial enhancement, transmural enhancement, and even a mixture of multiple enhancement patterns. The most common delayed enhancement pattern in the CA group was subendocardial linear enhancement, which was observed in 23 patients (76.7%), a significantly greater proportion than that in the HCM group (*P* < 0.001). In the HCM group, the most common delayed enhancement pattern was mid-myocardial patchy enhancement, which was observed in 17 patients (58.6%), also a greater proportion than that in the CA group, although the difference was not statistically significant (*P* = 0.091).

**Figure 2:**
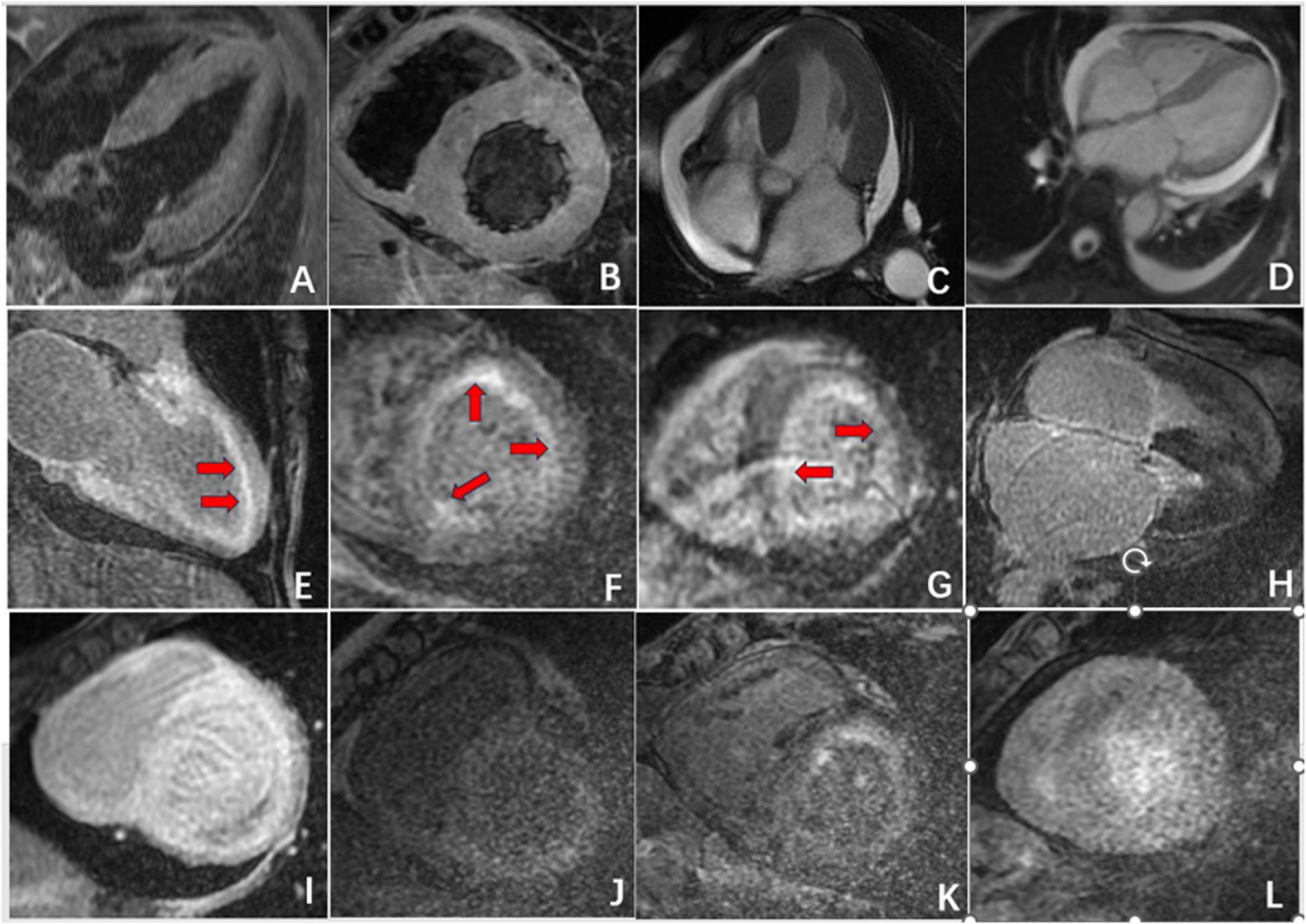
Radiographic findings of CA. **(A,B)** Symmetric myocardial hypertrophy; **(C,D)** pleural effusion and pericardial effusion; **(E,F)** subendocardial enhancement (red arrow); **(G)** transmural delayed enhancement pattern indicating diffuse myocardial infiltration; **(H,I)** delayed enhancement affected the left atrium, right atrium, and right ventricle; **(J,L)** “dark blood pool imaging” and “chaotic sign”.

**Figure 3:**
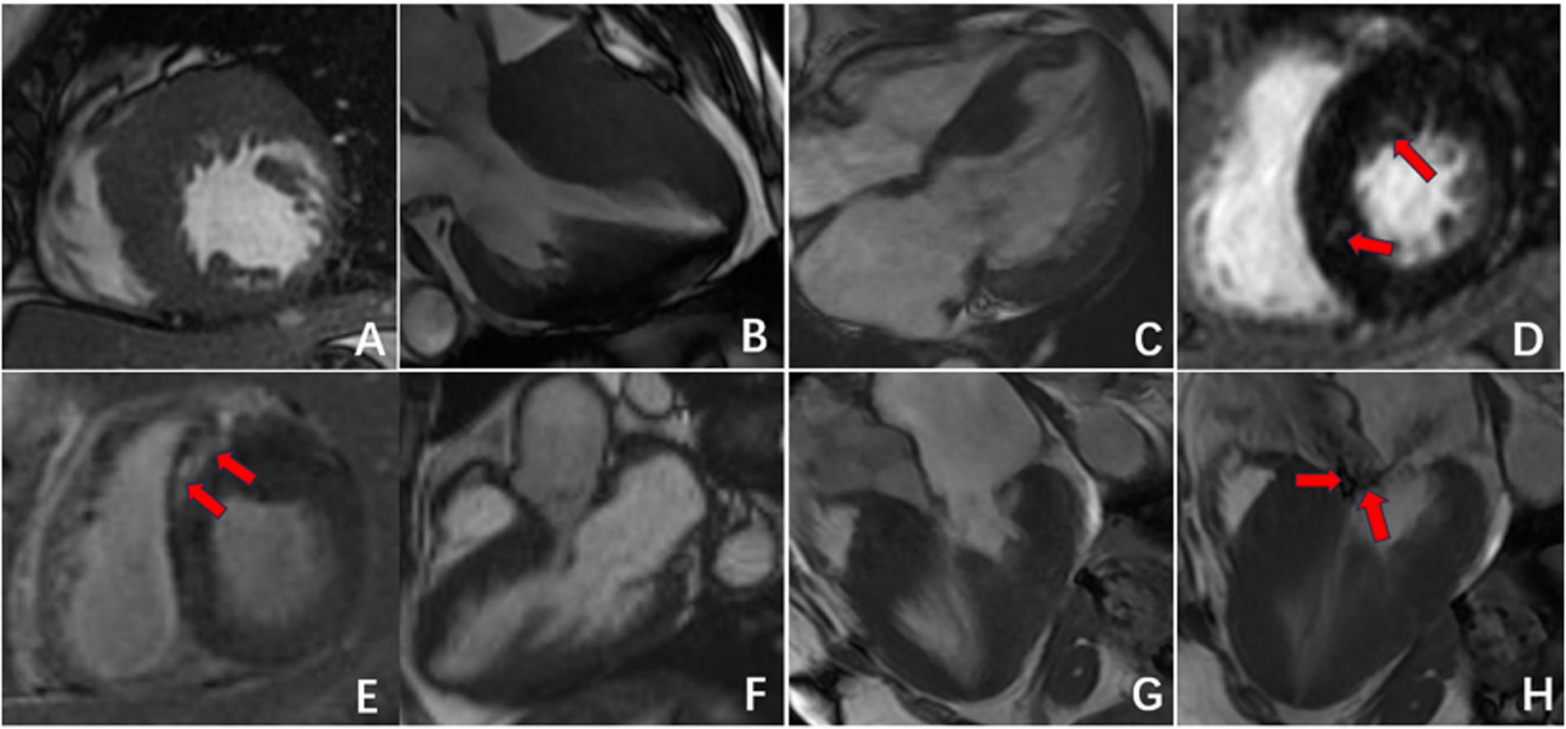
Radiographic findings of HCM. **(A–C)** Asymmetric myocardial hypertrophy; **(D,E)** mid-myocardial enhancement (red arrow); **(F–H)** HCM resulting in signs of an outflow tract with a systolic high-velocity blood flow signal (red arrow).

Furthermore, in 15 patients (50%) in the CA group, delayed enhancement not only affected the left ventricular myocardium but also involved the left atrium, right atrium, and right ventricle. In contrast, delayed enhancement in the HCM group did not extend to other cardiac chambers. In 10 patients (33.3%) in the CA group, a “dark blood pool imaging” phenomenon was observed, in which the signals of the blood pool and myocardium were indistinguishable after delayed enhancement, presenting a typical “chaotic sign”. This feature has specific diagnostic value for CA (Table 2, Figure 2, Figure 3).

**Table 2:** Comparison of delayed enhancement patterns and extent.

Variable	CA (*n* = 30)	HCM (*n* = 29)	*P*
No enhancement	6 (20%)	12 (41.4%)	0.075
Subendocardial enhancement	23 (76.7%)	1 (3.4%)	<0.001
Mid-myocardial enhancement	11 (36.7%)	17 (58.6%)	0.091
Transmural enhancement	8 (26.7%)	2 (6.9%)	0.094
Other heart chambers are enhancemented	15 (50%)	0	<0.001
Dark blood pool phenomenon	10 (33.3%)	0	<0.001

CA, cardiac amyloidosis; HCM, hypertrophic cardiomyopathy.

### Comparison of global myocardial strain parameters

As illustrated in Figure 3, the left ventricular GRS, GCS, and GLS parameters in both the CA and HCM groups showed varying degrees of reduction compared with those in the NC group, with statistically significant differences (*P* < 0.05). Further pairwise analyses revealed significant differences in GRS and GCS between the CA and HCM groups and between the CA group and the NC group (*P* < 0.05). However, there was no significant difference in these parameters between the HCM group and the NC group. With respect to GLS parameters, significant differences were observed between the CA and HCM groups, the CA group and the NC group, and the HCM group and the NC group (*P* < 0.05) (Table 3).

**Table 3:** Comparison of global myocardial strain parameters.

Variable	CA	HCM	NC	*P*
GRS	27.05 ± 16.07^a^^,^^c^	35.60 ± 13.77^b^	41.46 ± 8.24	0.001
GCS	−12.74 ± 9.79^a^^,^^c^	−17.82 ± 7.36^b^	−20.07 ± 2.80	0.003
GLS	−6.11 ± 5.90^a^^,^^c^	−9.96 ± 3.50^b^^,^^c^	−13.89 ± 3.62	<0.001

CA, cardiac amyloidosis; HCM, hypertrophic cardiomyopathy; NC, healthy control; GRS, global radial strain; GCS, global circumferential strain; GLS, global longitudinal strain.

a *P* < 0.05 vs. HCM group.

b *P* < 0.05 vs. CA group.

c *P* < 0.05 vs. NC group.

### Myocardial strain in ROC curve analysis of CA and HCM

**Figure 4:**
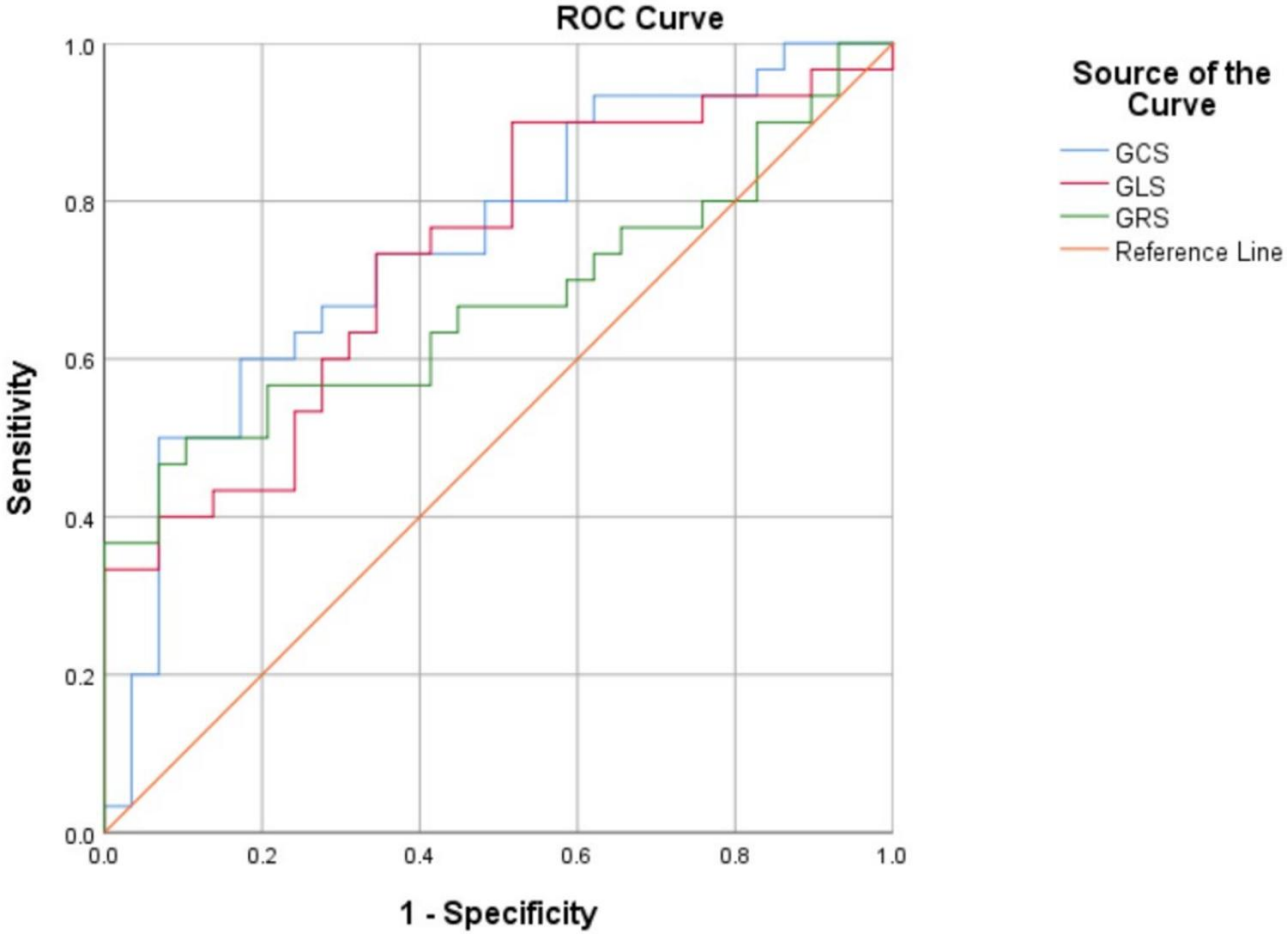
Receiver operating characteristic curves. global radial strain (GRS), global circumferential strain (GCS) and global longitudinal strain (GLS) between the cardiac amyloidosis (CA) and hypertrophic cardiomyopathy (HCM) groups.

The above findings suggested that GRS, GCS, and GLS have diagnostic value in discriminating between the CA and HCM groups. Further ROC analysis indicated that GCS (AUC = 0.748, 95% CI: 0.622–0.874; *P* = 0.001) and GLS (AUC = 0.732, 95% CI: 0.604–0.860; *P* = 0.002) had better diagnostic efficiency than GRS (AUC = 0.671, 95% CI: 0.530–0.813; *P* = 0.024) (Table 4, Figure 4). The optimal cutoff for GCS was −14.6%, yielding a sensitivity of 50% and a specificity of 93%; for GLS, the optimal cutoff was −9.22%, yielding a sensitivity of 73% and a specificity of 66%. AUCs are presented with 95% CIs in Table 4.

**Table 4:** Myocardial strain ROC curve analysis of CA and HCM.

Variable	AUC (95% CI)	Cut-off	Sensitivity	Specificity	*P*
GRS	0.671 (0.530–0.813)	78.17	0.47	0.93	0.024
GCS	0.748 (0.622–0.874)	−14.6	0.5	0.93	0.001
GLS	0.732 (0.604–0.860)	−9.22	0.73	0.66	0.002

GRS, global radial strain; GCS; global circumferential strain; GLS; global longitudinal strain; AUC, area under the ROC curve.

## Discussion

The present study demonstrated that CMR delayed enhancement patterns combined with myocardial strain analysis can effectively differentiate CA from HCM. Specifically, CA patients exhibited characteristic subendocardial or transmural delayed enhancement, more pronounced reductions in GLS and GCS compared with HCM and controls, and a higher prevalence of pericardial/pleural effusion. ROC analysis further confirmed that GLS and GCS had superior diagnostic efficiency over GRS in distinguishing CA from HCM. Unlike prior reports that evaluated single-parameter performance, our study integrates delayed-enhancement characteristics and quantitative strain metrics into a unified CMR diagnostic framework, thereby providing a more comprehensive and clinically applicable approach. These findings suggest that multiparametric CMR provides a valuable noninvasive approach for accurate differential diagnosis.

Both CA and HCM can present with left ventricular myocardial hypertrophy and increased mass, but their underlying pathophysiology and imaging features are distinct. CA occurs primarily due to diffuse deposition of amyloid substances in the left ventricle, leading to symmetrical and uniform thickening of the myocardium (14). In contrast, HCM is characterized predominantly by asymmetrical hypertrophy of the left ventricular myocardium, especially hypertrophy of the interventricular septum myocardial cells and fibrosis in the hypertrophic myocardial area (15–17), sometimes resulting in signs of outflow tract narrowing and obstruction (18). Additionally, CA patients often have pleural effusion or pericardial effusion, which may be related to heart failure or amyloid substance deposition (19). These overlapping clinical manifestations contribute to a high rate of misdiagnosis, underscoring the value of advanced imaging techniques for differentiation.

The results of this study indicate that delayed enhancement CMR patterns can serve as a useful method for distinguishing between CA and HCM. The enhancement pattern of CA is characterized mainly by subendocardial diffuse enhancement and transmural enhancement, findings that have been widely described in previous studies of cardiac amyloidosis using late gadolinium enhancement (20, 21). This enhancement pattern may be related to the deposition pattern of amyloid substances, which initially deposit subendocardially with blood flow distribution and progressively involve the entire myocardium, manifesting as transmural enhancement as the disease progresses. Additionally, when amyloid substances are deposited in other cardiac chambers, they can manifest as enhancement of varying degrees in the left atrium, right ventricle, and right atrium. Some researchers have proposed that the delayed enhancement imaging features of CA are related to the deposition of amyloid substances and thus correspond to CA prognosis (22–24). In contrast, delayed enhancement in HCM presents mainly as patchy enhancement in areas of hypertrophic myocardium, especially in the middle layer of the interventricular septum. In patients with CA, a distinctive phenomenon, termed the “chaotic sign”, is frequently observed on delayed enhancement images. We define this sign as a loss of normal contrast between the blood pool and myocardium, producing a diffusely hypointense ventricular cavity with blurred endocardial borders. This appearance reflects markedly altered gadolinium kinetics due to diffuse amyloid infiltration and rapid clearance of contrast from the blood pool. Similar “dark-blood” or “non-nullable myocardium” findings have been described in previous studies of cardiac amyloidosis (25, 26). Left ventricular systolic and diastolic deformation is achieved through the combined action of the three layers of myocardial fibers: subendocardial myocardium, mid-myocardium, and subepicardial myocardium. Each layer of myocardial fibers affects strain in different directions according to the angle of orientation (27, 28). The subendocardial myocardium, which is parallel to the long axis of the heart, affects mainly longitudinal strain, whereas the mid-myocardial fibers, oblique to the heart's outer surface, affect mainly circumferential strain; radial strain is affected by the combined action of all myocardial layers (29). In CA patients, amyloid proteins are deposited in the subendocardial myocardium in the early stages of the disease, predominantly affecting longitudinal strain. Therefore, the decreased longitudinal strain of the left ventricle is the most sensitive indicator for evaluating myocardial amyloidosis (30, 31). The results of this study indicate that, compared with the HCM and control groups, the CA group exhibited significantly reduced GLS, suggesting more pronounced damage to the subendocardial longitudinal myocardium in CA patients. Additionally, the GRS and GCS values in CA patients were significantly lower than those in the HCM and NC groups. The variation in these parameters may suggest that amyloid substance deposition gradually affects the mid-myocardium and subepicardial myocardium, indicating further deterioration in CA.

ROC analysis in this study showed that GCS (AUC = 0.748) and GLS (AUC = 0.732) had better diagnostic efficiency than GRS (AUC = 0.671) for distinguishing CA from HCM. This finding is consistent with the results of Oda and Williams. In Oda's study (32), with delayed enhancement as the gold standard, GCS achieved sensitivity, specificity, and accuracy values of 93.8%, 88.5%, and 89.2%, respectively, suggesting that GCS has high diagnostic value. In Williams' study (33) of CA and HCM patients, GLS differed significantly between the two groups, supporting our results. However, our findings differ from Jung (34), who reported that GRS had the highest accuracy (AUC = 0.898). This discrepancy may be attributed to differences in disease stage distribution across study cohorts: since GRS reflects global deformation integrating all myocardial layers, it may be more preserved in early CA but impaired in later stages, thus reducing its discriminative power in heterogeneous samples. Moreover, recent work by Zaarour et al. further supports the diagnostic value of strain analysis in differentiating CA from HCM, demonstrating comparable accuracy of GLS and GCS in a multicenter cohort (35). Our findings are consistent with their observations and additionally highlight that integrating strain indices with delayed enhancement patterns may enhance diagnostic confidence and clinical applicability.

In summary, the novelty of our work lies in the multiparametric integration of CMR-TT and LGE findings, the establishment of reproducible strain-based thresholds verified by ROC analysis, and the demonstration of their complementary diagnostic roles. By reinforcing methodological clarity and statistical transparency—including the addition of inter- and intra-observer ICCs and 95% confidence intervals for AUC values—we aimed to enhance the robustness and clinical relevance of our conclusions.

From a clinical perspective, these findings emphasize that CMR delayed enhancement combined with strain analysis provides a noninvasive and comprehensive method for differentiating CA from HCM. Accurate differentiation is critical because treatment strategies differ fundamentally: CA patients may benefit from systemic therapies such as chemotherapy or targeted amyloidosis treatments, whereas HCM patients are often managed with beta-blockers, septal reduction therapy, or implantable cardioverter-defibrillators. Misdiagnosis may lead to inappropriate therapy and worsen prognosis; studies have reported a high mortality in untreated CA, with median survival as short as 6–12 months in advanced cases (3). Therefore, the ability of CMR to identify specific patterns of myocardial involvement and quantify functional impairment may support earlier diagnosis, more accurate risk stratification, and optimized therapeutic strategies, ultimately improving patient outcomes.

Furthermore, although CA and HCM can overlap morphologically in “borderline” or grey-zone phenotypes, their risk stratification and prognostic assessment differ substantially. In HCM, validated sudden cardiac death prediction tools, such as the HCM-Risk-SCD score, are widely applied to guide implantable cardioverter-defibrillator (ICD) implantation and assess arrhythmic risk. In contrast, in CA, overall mortality is driven mainly by restrictive hemodynamics, conduction block, and heart failure progression rather than malignant ventricular arrhythmias, and the benefit of ICD therapy remains limited (36, 37). Recently, amyloidosis-specific staging and prognostic models integrating circulating biomarkers (NT-proBNP, troponin) and imaging indices have been developed to better predict disease progression and therapeutic response (38). These discrepancies highlight the importance of disease-specific risk frameworks when evaluating patients presenting with left ventricular hypertrophy of uncertain etiology, as conventional HCM algorithms may not be transferable to infiltrative cardiomyopathies such as CA.

In addition, endurance exercise can induce cardiac remodeling—often referred to as “athlete's heart”—which may mimic early or phenotype-positive HCM and create diagnostic grey zones. Differentiating between physiological adaptation and pathological hypertrophy is therefore clinically important, particularly in highly trained individuals who exhibit increased wall thickness or borderline strain values. In such cases, CMR provides valuable tissue characterization through strain mapping and parametric imaging, allowing the detection of subtle myocardial fibrosis, abnormal strain gradients, or extracellular-volume expansion that are absent in physiological hypertrophy (39). Recognizing these exercise-related phenotypes enhances diagnostic precision and prevents misclassification of benign athletic adaptation as cardiomyopathy.

To enhance clarity and clinical applicability, the distinguishing characteristics between CA and HCM are summarized in Table 5. This table presents a comparative overview of key morphological findings, myocardial strain patterns, risk-stratification features, and exercise-related diagnostic considerations, serving as a concise reference for differential diagnosis.

**Table 5:** Summary of diagnostic, morphological, and imaging differences between cardiac amyloidosis (CA) and hypertrophic cardiomyopathy (HCM).

Feature	Cardiac amyloidosis (CA)	Hypertrophic cardiomyopathy (HCM)
Myocardial hypertrophy pattern	Symmetric and concentric wall thickening due to diffuse amyloid infiltration	Asymmetric (septal-predominant) hypertrophy caused by myofibrillar disarray and fibrosis
Delayed-enhancement (LGE) pattern	Subendocardial or transmural enhancement; may involve atria or RV; characteristic “chaotic sign” and dark-blood-pool appearance	Patchy or mid-wall enhancement, typically localized to hypertrophic segments of the interventricular septum
Myocardial strain profile	Markedly reduced GLS and GCS with apical-sparing pattern; uniform reduction across layers as disease progresses	Regional strain heterogeneity; GLS relatively preserved except in hypertrophic segments
Left-ventricular mass and geometry	Increased LV mass with preserved cavity size; concentric remodeling	Increased LV mass with asymmetric geometry; may cause LV outflow obstruction
Extracardiac/associated findings	Pericardial or pleural effusion common; systemic amyloid deposition in other organs	Usually absent; no extracardiac involvement
Risk-stratification paradigm	Mortality driven by restrictive physiology, conduction block, and HF progression; ICD benefit limited; staging incorporates NT-proBNP, troponin, and CMR indices (36, 38)	Sudden-cardiac-death risk predominates; ICD decisions guided by HCM-Risk-SCD score (37)
Exercise-related phenotype	Not phenotype-driven; excessive exercise may worsen HF symptoms	Endurance training may mimic early HCM (“athlete's heart”); CMR strain and mapping help differentiation (39)
Prognostic outlook	Progressive diastolic dysfunction and restrictive failure; median survival 6–12 months if untreated	Generally favorable with appropriate management; sudden death preventable with ICD therapy

This table provides a qualitative summary derived from the present study's findings and cited literature. No new or unpublished data were introduced.

This study has several limitations. First, the sample size was relatively small and single-center. Second, we analyzed heterogeneous CA subtypes together (AL *n* = 25; ATTR *n* = 5) due to limited numbers, which may introduce confounding. Third, although endomyocardial biopsy remains the reference standard, not all CA patients were biopsy-proven. Finally, reproducibility (ICC) was not assessed because each strain parameter was measured once; future prospective studies should incorporate repeated measurements and multi-reader analyses. Future large-scale and multicenter studies are warranted to validate these findings and explore the prognostic significance of CMR strain parameters in CA and HCM.

## Conclusion

CMR can comprehensively assess myocardial hypertrophy, function, strain parameters, and delayed enhancement features, providing valuable diagnostic information. In particular, delayed enhancement patterns combined with GLS and GCS, with optimal cutoff values of −14.6% and −9.22%, can effectively differentiate CA from HCM, offering a noninvasive imaging approach with important clinical implications for guiding treatment and improving prognosis.

## Data Availability

The original contributions presented in the study are included in the article/Supplementary Material, further inquiries can be directed to the corresponding author.
